# Assessment of aortic stenosis severity by rest CMR correlates well with stress echocardiography in the setting of low left ventricular flow states

**DOI:** 10.1186/1532-429X-16-S1-P264

**Published:** 2014-01-16

**Authors:** Nav Chahal, Miguel S Vieira, Raad Mohiaddin

**Affiliations:** 1 Cardiovascular MRI, Royal Brompton Hospital, London, UK; 2 National Heart & Lung Institute, Imperial College, London, UK

## Background

Identifying the high-risk subset of patients with severe aortic stenosis (AS) and low transvalvular pressure gradient due to significant left ventricular (LV) impairment has relied on the assessment of the haemodynamic changes in response to flow normalization using pharmacological stress. Although cardiovascular magnetic resonance (CMR) aortic valve area (AVA) derived from planimetry has shown good correlation with rest echocardiography (RE) assessment [1], there is scant data on the correlation between rest CMR and stress echo (SE) for measurement of AVA in this cohort of patients.

## Methods

A total of 46 patients who underwent CMR and both RE and SE were retrospectively studied. Stress echo was clinically indicated in patients with suspected low-flow, low-gradient AS with preserved or impaired LV EF. CMR AVA was determined using planimetry, from the continuity equation during RE and SE. Cardiac index was derived from a through plane flow mapping sequence at the level of the sinotubular junction. Statistical analysis was performed using paired Student's t-test (SPSS version 21).

## Results

The mean age of patients studied was 77 years (SD 8 years). RE vs. SE values for mean gradient were 28 mmHg v 39 mmHg and for EF were 43% v 51% (both p < 0.001). In the whole cohort, mean AVA was similar in RE and CMR (0.91 cm^2 ^and 0.94 cm^2 ^respectively, p = 0.17), and significantly higher during SE (1.1 cm^2^) compared to both RE and CMR (p < 0.001 and p = 0.002, respectively). Differences in AVA between the modalities after stratification by EF and by flow rate are presented in table 1 and table 2, respectively. In patients with low EF, although higher by CMR, there was no significant difference in AVA when compared to RE. In low EF, AVA was similar between SE and CMR. However, in the presence of low flow rate, CMR AVA was significantly higher than RE AVA, but similar to SE AVA. In preserved EF and normal flow rate groups, there were no significant differences between RE and CMR AVA - however, SE AVA remained significantly higher than CMR AVA.

**Table 1:** Aortic valve area stratified by ejection fraction

Rest Echo EF < 40% (n = 20)			Rest Echo EF > 40% (n = 26)		
CMR AVA	RE AVA	SE AVA	CMR AVA	RE AVA	SE AVA

0.95 cm^2^	0.88 cm^2^*	0.95 cm^2^	0.94 cm^2 ^**	0.91 cm^2 ^†	1.1 cm^2^

* RE v SE p < 0.001; ** SE v CMR p = 0.002; † RE v SE p < 0.001 EF = ejection fraction, AVA = aortic valve area, CMR = cardiovascular magnetic resonance, RE = rest echo, SE = stress echo

**Table 2:** Aortic valve area stratified by cardiac index

CMR C.I. < 2.5 L/min (n = 28)			CMR C.I. > 2.5 L/min (n = 18)		
CMR AVA	RE AVA	SE AVA	CMR AVA	RE AVA	SE AVA

0.96 cm^2 ^*	0.87 cm^2 ^**	0.95 cm^2^	0.94 cm^2 ^†	0.91 cm^2 ^‡	1.1 cm^2^

* RE vs CMR p = 0.01; ** RE vs SE p < 0.001; †CMR vs SE p = 0.004; ‡ RE vs SE p < 0.001 C.I. = cardiac index, remaining abbreviations as table 1

## Conclusions

In patients with reduced flow rate, RE may over diagnose AS severity. There is a good correlation between rest CMR and SE in this group, suggesting that CMR planimetry of the aortic valve is adequate in these patients and the need for normalization of flow with pharmacological stress may not always be clinically necessary.

## Funding

None.
